# Grass is a tattletale: using grass as a biomonitoring tool for remote sensing of coal combustion residue contamination

**DOI:** 10.1007/s10661-025-14719-7

**Published:** 2025-12-09

**Authors:** Alice Goldstein-Plesser, Anna Ulanova, Maxwell Lutz, Julie Parno, Nicole Wuerslin, Korynna Rankin, Jazmine Hawkins, Margaret Kurth, Samuel Beal, Timothy Cary, Jeffrey Summers, Taylor Rycroft, Franz J. Lichtner

**Affiliations:** 1 Cold Regions Research and Engineering Laboratory, U.S. Army Engineer Research and Development Center, 72 Lyme Road, Hanover, NH 03755 USA; 2 Oak Ridge Institute for Science and Education, 1299 Bethel Valley Rd, Oak Ridge, TN 37830 USA; 3 Office of Fossil Energy and Carbon Management, U.S. Department of Energy, 1000 Independence Avenue SW, Washington, DC 20585 USA; 4 Environmental Laboratory, U.S. Army Engineer Research and Development Center, 3909 Halls Ferry Rd, Vicksburg, MS 39180 USA

**Keywords:** Biomonitoring, Coal combustion residuals (CCR), Unmanned aerial vehicle (UAV), Spectral imaging, Grass bioindicator

## Abstract

Coal-fired power plants generate coal combustion residuals (CCRs), which are typically disposed of in landfills and surface impoundments that must be monitored to ensure that hazardous constituents such as arsenic (As) and selenium (Se) do not escape into the surrounding environment. Traditional methods for monitoring surface impoundments, such as sampling discharge of all outlets of hydraulic structures to approximate CCR concentrations, are highly resource-intensive and largely manual, posing a significant cost challenge for the stakeholders responsible for them. The use of grass species as bioindicators may offer a more efficient and sustainable method for long-term monitoring of CCR impoundments. In this study, we sought to determine whether three species of grass could serve as effective bioindicators for detecting changes in As and Se soil contamination profiles and provide an evaluation of three technologies used for evaluating plant health. *Lolium perenne*, *Panicum virgatum*, and *Paspalum notatum* were treated with As or Se and monitored with a spectroradiometer and multispectral camera to detect a spectral response to the chemical stress. Metal transfer analysis revealed that there was a significant change in metal concentrations in plant tissue across the grass species and different treatments, with *Paspalum virgatum* providing the best dose response after a week of Se treatment. *Lolium perenne* provided a response past the Se toxicity threshold of 5 mg/kg. We then expanded this study to a field scale to determine if our results would translate to an environmentally relevant scale. Unmanned aerial vehicle (UAV) results suggested *Lolium perenne* was efficient in determining whether CCR was present in soils but lacked sensitivity to differentiate between low and high loadings. Monthly sampling also revealed that metal concentration in plant tissue decreased as plants underwent senescence. Data collected with the UAV proved to be the most proficient method of determining a dose response.

**Supplementary Information:**

The online version contains supplementary material available at 10.1007/s10661-025-14719-7.

## Introduction

Approximately 38% of power is generated by coal worldwide, with the USA ranked among the top three nations for coal combustion (Zierold & Odoh, 2020). US utility-scale net electricity generation by coal in 2023 was 16.2% according to the Energy Information Agency (U.S. EIA, 2024). Coal-fired power plants produce a waste stream of coal combustion residuals (CCRs), which are typically disposed of in landfills and surface impoundments (Deonarine et al., 2023; Kravchenko & Ruhl, 2021). CCRs include fly ash, bottom ash, boiler slag, and material from flue gas desulfurization (Zierold & Odoh, 2020). CCRs can contain potentially toxic metals and other compounds that are persistent, bioaccumulate, and toxic, posing potential human and ecological health risks if containment structures leak or rupture (Haskins et al., 2017; Pudasainee et al., 2020). High concentrations of selenium (Se) and arsenic (As) in CCRs are of particular concern due to their toxicity and their ability to bioaccumulate in the environment (Fu et al., 2018; Zwolak, 2020). Both As and Se can be carcinogenic, cytotoxic, and genotoxic, and at low concentrations, Se may also enhance As toxicity (Huang et al., 2019; Sun et al., 2021).

The behavior of selenium (Se) and arsenic (As) in soils is strongly influenced by soil composition. The release of both metals into the soil is primarily governed by adsorption and metal complexation processes (Garcia-Manyes, 2002; Tolu et al., 2014; Yan et al., 2025), which are affected by factors such as the presence of competing anions, clay minerals, iron oxides, and aluminum compounds (Cai et al., 2003). Among selenium species, selenite tends to bind more readily to surface soils than selenate; however, selenate is generally more mobile and bioavailable for plant uptake (Cai et al., 2003; R. Li et al., 2010a, b). Likewise, As fate and transport depend on the functional groups present in dissolved organic matter, with carboxyl and hydroxyl groups result in higher adsorption on goethite surfaces and mobilization (Yan et al., 2025).

The US Environmental Protection Agency (U.S EPA, 2015) regulates the disposal of CCRs in landfills and surface impoundments to ensure they are properly managed and do not pollute waterways, groundwater, drinking water, and air. In April 2024, the EPA expanded its national minimum requirements to include inactive or legacy surface impoundments, which are more likely to be unlined, unmonitored, and therefore considered more prone to leaks and structural problems than units that are currently in service (U.S. EPA, 2024). Traditional methods for monitoring surface impoundments (e.g., installing monitoring wells and regularly sampling groundwater) are highly resource-intensive and largely manual, posing a significant cost challenge for power facilities and grid operators with responsibility for these newly regulated inactive surface impoundments. The considerable cost requirements of long-term monitoring, coupled with the significant human health and ecological hazards posed by CCRs should they leach into the environment, necessitate more efficient and sustainable methods for long-term monitoring of CCR impoundments and their surrounding environments.

One potential alternative for continuous and long-term monitoring of CCR impoundments is the use of plants as bioindicators (Cakaj et al., 2024). Bioindicators are living organisms that respond to environmental change in a manner that is detectable and interpretable. Numerous species, spanning all the biological kingdoms, have served as successful bioindicators. However, plants are uniquely suited for long-term, continuous, and remote monitoring of environments as they can be predictably relocated and can cover large areas of land. Grasses, in particular, are promising bioindicator species as they are already found covering large land masses or can easily be planted to cover a large area. Grasses are capable of living in a wide variety of environments and can create a spectral dose–response signal when exposed to certain contaminants (Carignan & Villard, 2002). Grasses have a variety of stress responses when exposed to environmental stressors – such as the heavy metals in CCR.

The main site for plant-metal interaction is in the root apical meristem, where the metal inhibits growth and disrupts typical root architecture formation (Angulo-Bejarano et al., 2021). Morphological disruptions are further driven by vascular system damage due to the presence of heavy metals and cell wall thickening (Chukwu & Gulser, 2025). Leaf chlorophyll concentration is a commonly used indicator of grass health since chlorophyll is the most abundant pigment found in photosynthetic cells and can change in abundance when plants are stressed (Singhal et al., 2019). The degree to which these changes—both morphological and chemical—occur can result in a dose-dependent response. This can alter the spectral signature of above-ground plant biomass, providing a multi-criteria response that can be read with spectral imaging technology (Lassalle et al., 2019). Grass root response to heavy metal stress has not been well studied (Joshi et al., 2022; Xu et al., 2016), though it clearly shows specific responses depend on contaminant type and plant species regulation. Choosing plant bioindicators must consider both above-ground and below-ground interactions as the field expands.

 Imaging spectrometers, such as multispectral cameras, collect spatially resolved spectral data in many narrow wavelengths throughout the visible and near-infrared portions of the electromagnetic spectrum (Bellante et al., 2013; Cheshkova, 2022). The spectral responses of plants to stress or changes in the environment can be captured using these devices. Thus, multispectral cameras have been mounted on greenhouse rails (Thomas et al., 2018) and on unmanned aerial vehicles (UAVs) (Neupane and Baysal-Gurel, 2021) to detect disease in agricultural plants. The resulting spectral data are often summarized using empirical vegetation indices, enabling the mapping of vegetation state related to specific physiological parameters (Barton, 2012; Lassalle et al., 2020).

To effectively use plants as bioindicators of CCR hazards, it is essential to understand the degree to which CCR, and the various toxic heavy metals within it, influence the spectral signature of plants. It is established that plants respond to the types of heavy metals present in CCR. Soybeans dosed with As exhibited a spectral shift of the chlorophyll absorption band, also known as the red edge (680 nm), to shorter wavelengths and higher reflectance in the 550–650 nm range (Milton et al., 1989). In contrast, soybean plants treated with Se had an inverse response, with the spectral position shifted to longer wavelengths and lower reflectance between 550 and 650 nm when compared with control plants (Milton et al., 1989). Bandaru et al. (2010) quantified the internal structural changes in the leaves of spinach plants coincident with spectral analysis (Bandaru et al., 2010).

The use of grasses as bioindicators of CCR is an unexplored and novel avenue. In our experiments, we are investigating if grass species can provide a signature reflectance as a dose response to increasing CCR concentrations. The experiments were broken into a small and controlled pot experiment and then continued in a field greenhouse to mimic more realistic seeding scenarios. The primary aim of the pot study was to use trace elements, Se and As, as a proxy to CCR to determine whether three species of grass could be used as bioindicators and if the grasses could display a signature signal for each treatment group. Successful bioindicators would display different signals when detecting changes in As and Se soil concentrations. Plants that have a well-established mechanism for phytoremediation are likely to be poor bioindicators, as they have adaptations to maintain their health.

Three different grass species were chosen to represent different climate types (cold and warm) as well as different carbon metabolism systems (C3 and C4 photosynthesis) (Gates et al., 2016; Lichtenthaler & Babani, 2022; Sage et al., 2015; Talukder & Saha, 2017). As and Se were chosen due to their highly toxic profile, representing trace CCR elements that have high environmental toxicology impacts. The field study utilized plots dosed with CCR to validate that the controlled pot experiment could be expanded to a real-world scale. The field study lasted for four months, allowing us to evaluate our technologies for the use of monitoring long-term CCR impoundment sites that require a minimum of eight samples for each background well in the first 6 months of sampling a new CCR impoundment site (U.S. EPA, 1979). By applying differing concentrations of CCR, we explored whether grasses can be used as a reliable bioindicator and different technologies that can provide efficient and reliable detection. We also established a data processing pipeline to analyze the spectral imaging used to detect heavy metal stress in the environment.

## Methods

### Grass cultivation

Pot experiments were conducted at the Cold Regions Research and Engineering Lab (CRREL) in Hanover, New Hampshire. Three grass species that have previously shown growth potential at ash impoundments in either cool or warm climates were selected: Perennial Ryegrass (*Lolium perenne*; cool climate adapted; hereafter referred to as “PRG”), Switchgrass “Carthage,” North Carolina Ecotype (*Panicum virgatum*; warm climate adapted; hereafter referred to as “SG”), and Argentine Bahia grass (*Paspalum notatum*; warm climate adapted; hereafter referred to as “Bahia”). Germination trials were conducted to calculate the seeding rate for each species. To first determine the germination rate for each species, a known number of seeds was sown on sterilized sand in standard 10 × 20 propagation trays. The trays were surface watered as needed and covered with 2.5″ humidity domes. Germination checks occurred weekly over the course of 3 weeks. A seed was considered germinated with the emergence of the cotyledon. The germination rate was calculated as the number of germinated seeds/number of seeds planted. The seeding rate in grams per square foot was then calculated using the following formula:

$$\% \;purity\; (provided\; on\; seed \;package) \times \% \;germination = pure \;live \;seed \;(PLS)$$

$$PLS/lbs\; per\; acre\; (provided\; on\; seed\; package) = seeding \;rate\; (lbs/acre)$$

$$\mathit{\left({lbs\;per\;acre\;\times\;grams/lb}\right)}\mathit/\mathit\;sq\mathit\;ft\mathit\;in\mathit\;acre\mathit\;\mathit=\mathit\;seeding\mathit\;rate\mathit\;\mathit(grams\mathit/ft^{\mathit2}\mathit)$$

Each species was planted into double-layered mesh trays (27.9 cm × 55.8 cm) which were lined with filter fabric to prevent the planting media from falling out while still allowing for drainage to occur.

To prepare for planting, each double-layered mesh tray was filled with 9 kg of sterilized, dried, and sieved (10 mm) sand (Figure S1). Three double-layered mesh 27.9 cm × 55.8 cm trays with sand were then placed into each large white containment tray (1.32 m × 0.71 m) in a randomized block (Figure S2). The block and the plant species location were randomly assigned. There were four replicates of each treatment for both Se and As experiments. Double-layered mesh trays within each containment system received 15 L of water. Trays sat for approximately 24 h before seeds were planted so the sand planting media was sufficiently moist for seed germination. Seeds were planted at a rate of 0.024 g/in^2^ (Bahia), 0.005 g/in^2^ (SG), and 0.0008 g/in^2^ (PRG). The weighed seeds were planted onto the double-layered mesh trays. The planting area of each double-layered mesh tray was divided into three even sections, where the middle section was left unplanted so Se or As treatments could be applied directly to this section once plants germinated and reached a mature stage. Clear propagation domes (Jiffy, 27.9 cm × 55.8 cm × 6.35 cm) were placed over the trays to aid germination and stayed until 2 weeks post-germination. Watering occurred three times per week, or as needed throughout the experiment, by a drip irrigation system emitting ~ 2.5 L in a 40-min watering session.

Field experiments were performed in 6.1 m × 6.1 m growth plots. A temporary greenhouse was constructed over the plots to control moisture content and limit large temperature fluctuations. The greenhouse roofing was constructed of a single layer of 0.1524 mm clear plastic, allowing for spectral collections to be taken of the plots without roof removal. Three treatment groups (control, low, high) were randomly assigned to a plot in the greenhouse with six replicates. Initially, Bahia grasses were seeded after analyzing the promising pot study results; however, Bahia failed to germinate due to the cool temperatures. PRG was ultimately chosen to seed the field plots due to its ability to grow in cooler climates since the field experiment took place in the fall months. Plots were seeded with 34.4 g/m^2^ of PRG seed.

### Contaminant application and sample collection

Se and As exposure experiments for the pot experiments were conducted separately between June and August 2023 and August to December 2023, respectively. Four different treatments were applied to the grasses: 0 mg/kg, 5 mg/kg, 15 mg/kg, or 25 mg/kg of sodium selenate (Thermo Scientific) or sodium arsenate (Thermo Scientific) solution.

The first sample collection for each treatment group took place prior to treatment. The first collection, the Se treatment group, took place 53 days after the seed planting date for all three grass species. In the As treatment group, collection number 1 took place 90 days after planting the Bahia and 74 days after planting the PRG and SG. To initiate exposure, 1 L of treatment solution was applied to the middle section of each mesh tray. The differing initial growth times for grasses to reach maturity were due to changes in seasonality between the Se and As experiments.

The experiment sample collections included collecting soil, above ground vegetation cuttings, and spectral measurements with two instruments (see the “Spectral data collection” section). A form of destructive sampling occurred for vegetation and planting-media sampling collections, where two samples were taken from each double-layered mesh tray to create a composite sample. No below ground plant biomass was collected. Recording of plant heights and taking spectral measurements were forms of non-destructive sampling. Sample collections that took place after application treatments of Se or As were as follows: sample collection number 2 was performed one day (24 h) after treatment, collection number 3 was performed seven days after treatment, and collection number 4 was performed 28 days after treatment. Sampling timepoints were selected to capture the initial plant stress response after dosing, the acclimation phase which can last several days, and a maintenance phase where plant homeostasis is maintained under stress conditions (Kosova et al., 2011). By temporal monitoring after 1, 7, and 28 days, the hyperspectral signal was monitored to see if the reflectance signal changed during these phases and confirm the method for long-term monitoring.

Field experiments were carried out at either control, “low,” or “high” levels of CCR (Longview Power, WV). Raw CCR metal contamination was analyzed by the Dartmouth Trace Element Analysis Core via ICP-MS (Supplemental Table 1). Each 6.1 × 6.1 m plot was divided into three, even sections (2.03 × 6.1 m), and CCR treatments were evenly spread across the growing area. “Low” treatments consisted of 3.66 kg/m^2^ and “high” treatments were 10.99 kg/m^2^. Treatments were covered with a 7.62 cm deep layer of sand and watered to lock in placement prior to seeding.

**Table 1:** One-way ANOVA of ICP-MS chemical analysis of metal accumulation in both soil and plant samples. * indicates a significant value <.05, ** < 0.005, and *** < 0.0005

Treatment	Substrate	Group	df	*F*-value	*p*-value
As	Soil	Time	2	2.40	0.0710
		Treatment Concentration	3	0.27	0.8454
		Species	3	0.93	0.3971
	Plant	Time	2	1.64	0.1980
		Treatment Concentration	3	5.38	0.0017**
		Species	3	21.45	< 0.0001**
Se	Soil	Time	2	13.94	< 0.0001**
		Treatment Concentration	3	27.01	< 0.0001**
		Species	3	1.89	0.1562
	Plant	Time	2	3.81	0.0120*
		Treatment Concentration	3	14.21	< 0.0001**
		Species	3	29.97	< 0.0001**

### Metal accumulation chemical analysis

Each pot experiment sample taken from the vegetation and planting media was ~ 0.5–1 g and dried in an oven at 75 °C for 16 h after collection for chemical analysis of As and Se concentration. Vegetation samples were then ground to 20 mm using a mill (Arthur H. Thomas Co.). Grasses collected during the UAVV experiment were cut with sterilized scissors to ~ 20 mm. Samples of both cut vegetation and planting media were then weighed for acid digestion.

Analysis of total As and total Se followed EPA Method 3050B (U.S. EPA, 1996), requiring homogenized soil and plant tissues to be acid-digested on a hot block, which captures metals that could become environmentally available. Briefly, a 500 mg sub-sample of each material was weighed to the nearest 0.1 mg in polypropylene tubes (SCP Science), and then a total of 5 ml trace metal grade HNO_3_ and 5 ml H_2_O_2_ were added over the course of repeated heating periods at up to 90 °C for 2 h. Digestates were diluted to 50 ml with type I water and then aliquoted and diluted for analysis. Pot experiment samples were analyzed by inductively coupled plasma mass spectrometry (ICP-MS) at the Dartmouth Trace Element Analysis (TEA) laboratory and by graphite furnace atomic absorption spectrometry (GFAAS; Perkin Elmer AAnalyst 600). Field experiment samples were analyzed by ICP-MS (Thermo iCAP TQe) in triple-quadrupole oxygen mode using mass 75 to 91 and 78 to 94 for As and Se, respectively. Instruments were generally calibrated with 5 to 9 points over the range of 0.05 to 100 μg/l using NIST-traceable standards (Inorganic Ventures), with associated detection limits in solid material of approximately 0.05 mg/kg. Calibration curves were verified with a NIST-traceable standard from a separate lot or manufacturer. ICP-MS analyses used an internal standard of Yttrium-89 or Indium-115. Quality control included method blanks, samples matrix-spiked to 10–20 mg/kg-sample, laboratory control samples spiked to 10–20 mg/kg-sample, and the standard reference materials NIST 2710a (Montana Soil), NIST 1547 (Peach Leaves), and NIST 1573a (Tomato Leaves). A method blank was included in each digestion, while the remaining QCs were run once per matrix.

Arsenic speciation in pot experiment samples was analyzed by liquid chromatography ICP-MS at the Dartmouth TEA lab. This analysis method separates the organic species monomethylarsonic acid (MMA), dimethylarsinic acid (DMA), and unretained organic species tentatively labeled as arsenobetaine (AsB), as well as the inorganic species As(III) and As(V). Soils were extracted with 0.1 M sodium hydroxide that has been shown to be an efficient extractant from iron oxide minerals but may result in some oxidation of As(III) to As(V) (Jackson and Miller, 2000). For these extractions, 10 ml of 0.1 M NaOH was added to 100 mg of soil, vortexed, and shaken for 4 h before being filtered at 0.45 μm. Plants were extracted with 0.28 M nitric acid following an established method for arsenic speciation in rice and rice products (FDA, 2012). Briefly, a 250 mg sub-sample of plant tissue was extracted with 10 ml of 0.28 M HNO_3_ with heating to 95 °C for 90 min, then diluted with 6.7 ml of deionized water, centrifuged, and filtered at 0.45 μm. Detection limits for As species in solid material were approximately 5 μg/kg.

Relative standard deviations (RSDs) from triplicate digestions of plants were 5% for As and 4% for Se. RSDs from triplicate digestions of soils were 4–23% for As and 25–37% for Se. Matrix spike recoveries of As and Se were 101–104% (*n* = 2) in plants and 86–117% (*n* = 4) in soil. LCS recoveries of As and Se were 97–101% (*n* = 2) in plants and 94–104% (*n* = 4) in soil. Recoveries from plant SRM certified values were 91–103% for As and 83–131% for Se (*n* = 6). Recoveries from soil/sediment SRM CLP median (2711a) and certified (1944) values were 80–102% for As and 75–99% for Se (*n *= 6).

### Spectral data collection

Spectral data collections were coincident with the sample collection dates, occurring just prior to treatment—once the plants were fully mature—and then 1, 7, and 28 days post-treatment. A spectroradiometer (HR-1024i field-portable spectroradiometer; SpectraVista Corporation (SVC)) and Headwall Co-aligned visible near infrared (VNIR) and shortwave infrared (SWIR) hyperspectral instrument (HSI) were used to characterize each plant’s spectral signature. Reflectance was determined with the SVC using the ratio of the radiance of the plant to the radiance of a calibrated 99% Spectralon white reference panel. Measurements were performed at both plant and blade scale. At the plant scale, two full-spectrum tungsten halogen lamps (ASD, Inc. Illuminator Lamps) were suspended over the tray for consistent lighting. The SVC was fitted with an 8-degree field of view (FOV) lens and mounted on a tripod with the lens placed 80 cm from the soil surface and approximately 70 cm above the estimated average plant height, resulting in a circular measurement area approximately 10 cm in diameter. For each double-layered mesh tray, a reference scan was taken followed by five scans of the tray, with small shifts in tray position for each scan such that a slightly different sample was within the FOV for each. This allowed for capture of any variability in plant health within the individual double-layered mesh tray. An SVC leaf clip and reflectance probe (LC-RP; SpectraVista Corporation) with an internal light source, combined with a fiber optic cable, was used to capture the reflectance at the blade scale. For each scan, several blades of grass were clipped into the device and scanned. For each double-layered mesh tray, a reference scan was completed followed by four blade scans.

### Spectral data processing

#### Spectroradiometer

The spectroradiometer data was preprocessed using the SVC HR-1024i PC Data Acquisition Software (version 1.22.26) and Python 3.8.19. The SVC overlap removal and detector matching algorithm was applied to each file. The scans received an initial quality control by plotting and removing any unacceptable scans, such as those that were near zero reflectance or visibly affected by any variable lighting conditions. The mean reflectance of each tray for each collection was calculated using Python along with the standard deviation and 25th and 75th percentiles to provide a sense of the variability among the averaged scans. The scans were then compiled and exported to R (version 4.3.3) (R Core Team, 2023) for further data exploration. Normalized differences of the spectrograms between the 0 and 25 mg/kg treatments were calculated, along with spectra visualizations of the different treatment types and species combinations. Principal component analysis (PCA) of spectroradiometer-derived vegetative indices was also conducted through the inclusion of on-site meteorological data to discern the driving forces of health degradation of the experimental grasses (Figure S3).

As similar process was completed in the field experiment. Cells C3 and C4 (Fig. 4) were chosen for SVC scanning on single leaf scale and plant scale. NDVI was calculated for the cells using an average for red wavelengths (620–670 nm) and NIR wavelengths (842–876 nm).

$$NDVI= \frac{({\mathrm{NIR}}_{842-876\text{ nm}}-{R}_{620-670\text{ nm}})}{({\mathrm{NIR}}_{842-876\text{nm }}+ {R}_{620-670\text{ nm})}}$$

Raw SVC data was plotted for control, low, and high groups across the wavelengths to see reflectance trends. All plotting was created using the R package ggplot2 (version 3.5.1) (Wickham, 2016).

#### Multispectral sensors

Grasses grown in the field greenhouse were analyzed for NDVI by using UAV drone (Harris Aerial Carrier Hx8) on October 23rd, 11 weeks after seeding, which is when the plants had at least two sets of true leaves. Flight procedures were as follows: speed of drone ~ 2 m/s, height over greenhouse was 39 m using both a Headwall Hyperspectral Co-Aligned HP (Headwall Photonics) sensor, and MicaSense Altum-PT (AgEagle Aerial Systems Inc.) multispectral sensor. Field greenhouse data was mosaicked by using the QgsRasterLayer feature from the core functionalities of QGIS (version 3.34.11) (QGIS Development Team, 2024). NDVI was calculated by using the Raster Calculator option of QGIS, using bands that correspond to red wavelengths (620–670 nm) and NIR wavelengths (842–876 nm). Three coordinate points were chosen for each section within the cells that would appear on all collection dates and were used as center points to create a cropped square of the calculated NDVI raster, resulting in a sampled 0.2 m^2^ area. The three squares for each section in a cell were used to calculate the average pixel value in the area as a proxy for average NDVI in the samples. The averages were calculated using the core QGIS capabilities such as QgsRasterLayer and QgsRasterBandStats. SciPy’s packages f_oneway and tukey_hsd were used to complete one-way ANOVA and the Tukey’s honestly significant difference (HSD) test (version 1.15.3) (Virtanen et al., 2020).

## Results and discussion

### As and Se concentration in soil and plant tissue

**Fig. 1:**
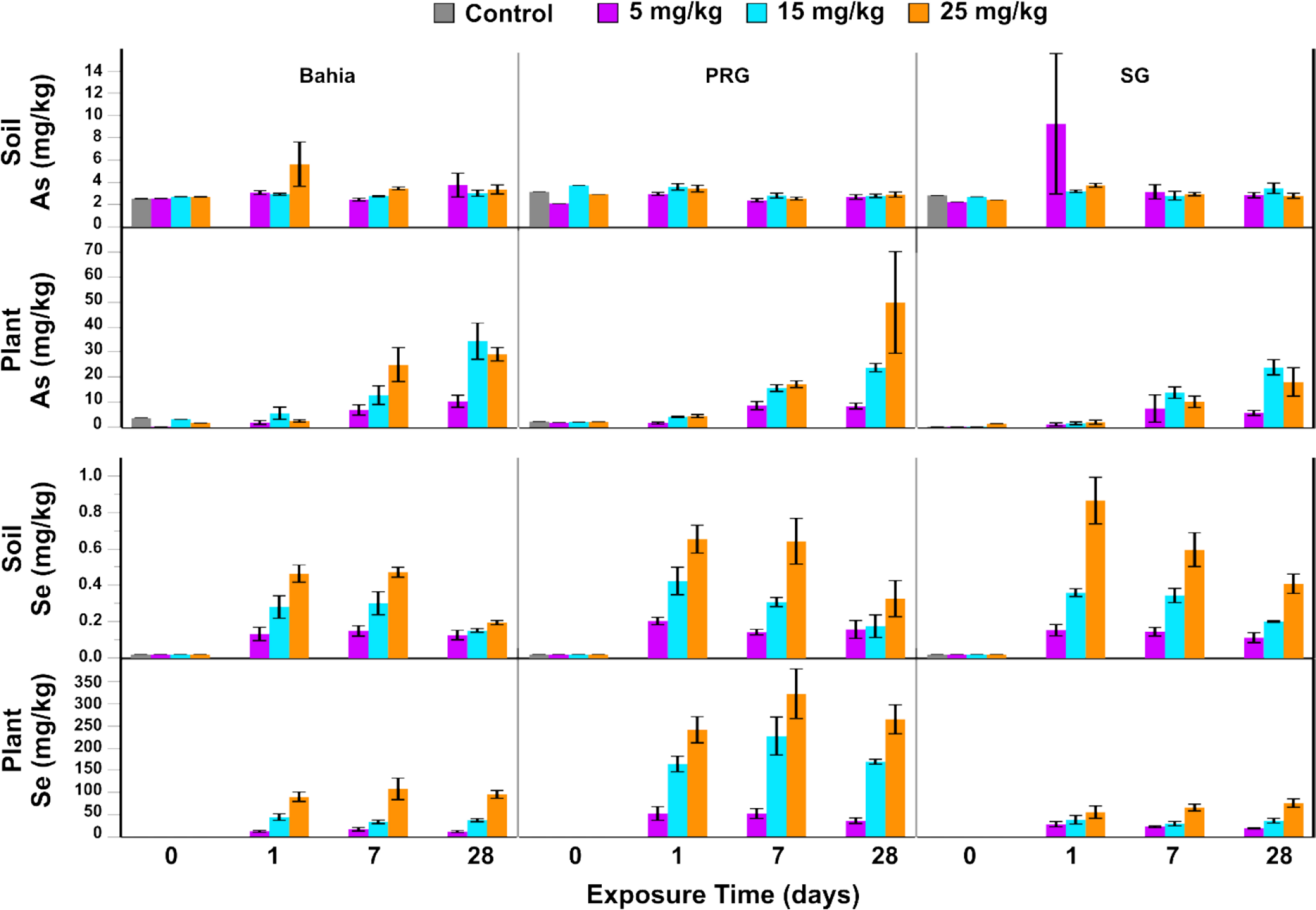
As and Se concentrations present in the soil and plant samples as determined by ICP-MS and GFAAS. Error bars represent standard error of four replicate measurements

Soil and above ground plant tissue were chemically analyzed for As and Se concentrations throughout the experimental timeline, with time 0 referring to collections taken of mature plants prior to treatment (Fig. 1). The concentrations of As and Se chosen (5, 15, and 25 mg/kg) represent environmentally relevant concentrations of As and Se contamination (reference concentrations of As = 1.94 mg/kg ± 1.65 mg/kg, Se = 4.93 mg/kg ± 2.27 mg/kg; CCR contaminated site concentrations of As = 33.59 mg/kg ± 15.35 mg/kg, Se = 9.87 mg/kg ± 3.34 mg/kg) when compared to field studies surrounding coal combustion containment facilities (Adriano et al., 1980; Holland et al., 2025). Soil As and Se content was monitored to both confirm the presence of As and Se at the dosed concentrations and confirm that containment integrity was not compromised or that there was no unintended dispersion of contaminants through loss through successive water treatments. Plant As and Se concentrations were monitored to assess the relative transfer of metals into the plant tissue.

**Fig. 2:**
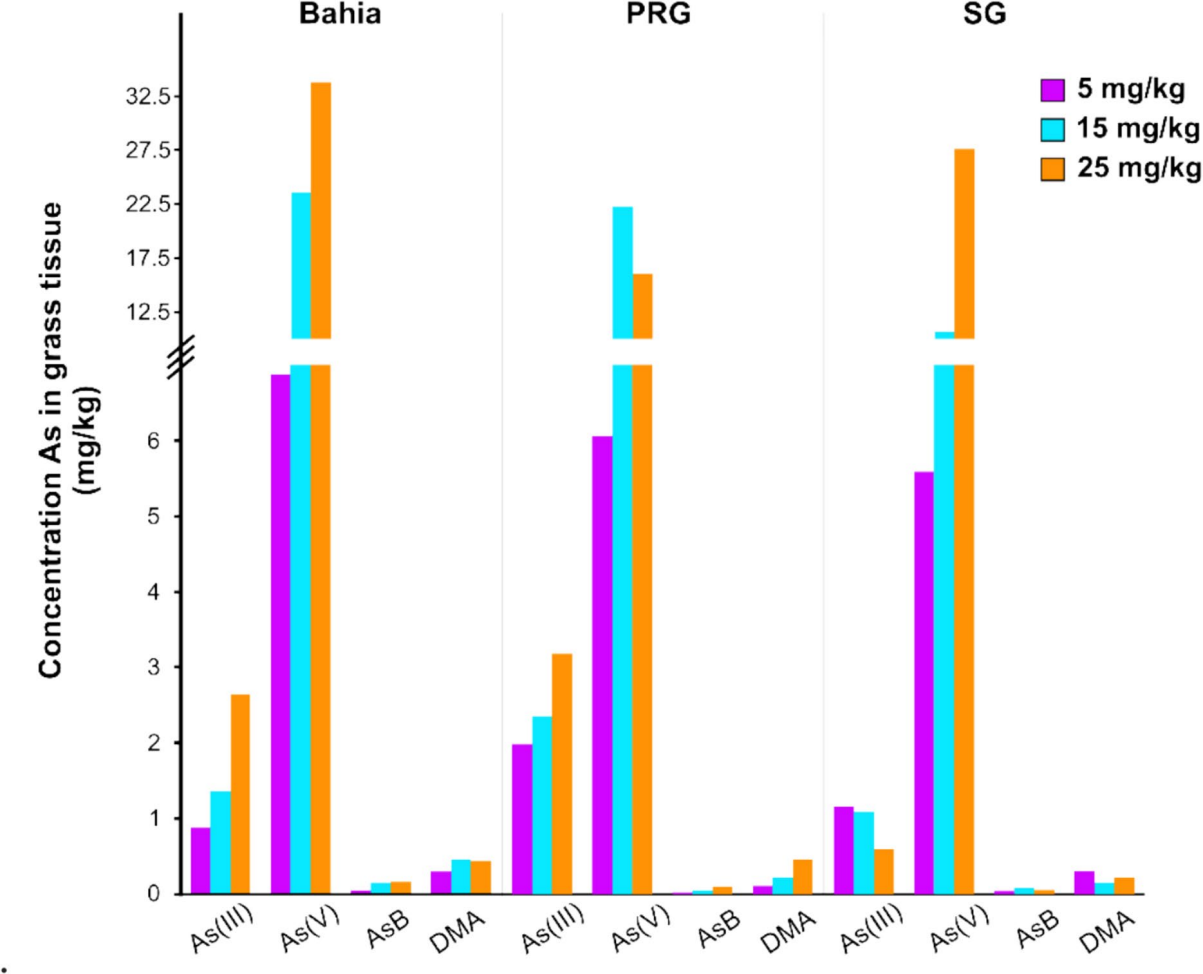
As speciation in pot study plants 28 days post exposure. Values represent four plant samples pooled into one ICP-MS run

Analysis of As-treated soils by ICP-MS showed no significant difference in As concentrations across all treatment concentrations or plant species (Table 1). This is not entirely surprising given the natural occurrence of As in mineral deposits in non-contaminated soils can naturally range from 0 to 100 mg/kg (Walsh et al., 1977). Therefore, the concentration of As of 2 mg/kg in control groups across all grass types indicates that there was an As concentration in the soil substrate used for all pot study experiments. Despite the soil results, As uptake and accumulation increased in a dose-dependent manner over the course of the first 7 days across all plant species (Fig. 1). These results suggest the As present in the control soils was not bioavailable for plant uptake and obscured the spike concentration of sodium arsenate. The As treatment in the form of arsenate (As(V)) is readily taken up by plants and likely led to the observed dose response in plant tissue (Fig. 2). Despite no significant differences in pairwise comparisons of each species at each time point (data not shown), there is a significant difference between the average accumulation of As across all species (*f* < 0.0001) (Table 1). This difference may be explained as Bahia and SG—residing in the same subfamily and more closely related to each other—are known for being more drought and heat tolerant than PRG. Advantages to tolerating abiotic stresses, such as heat and drought, can be linked to a cross-tolerance in other stressors such as metal toxicity (Hossain et al., 2016).

Control soils all had Se concentrations below the detection limit of 40 μg/kg (Fig. 1). The application of sodium selenate increased Se concentrations in a dose-dependent manner at 24 h of exposure; however, these concentrations were far lower than the dosed concentrations of 5, 15, and 25 mg/kg (Fig. 1). The relatively low concentrations of Se in soil fractions suggest that Se was retained by adsorption of aqueous Se, and the subsequent decrease over time was due to desorption modulated by both the plant and abiotic (temperature) environment. Soil concentrations of Se significantly changed over time (*p* < 0.0001) and by treatment concentration (*p* < 0.0001) (Table 1). Interestingly, the average concentration of Se in Bahia soils from the 25 mg/kg treatment group was significantly lower than PRG and SG soils after 24 h (Kruskal–Wallis test; *p*-value = 0.0498). Average Se levels in Bahia soils from the 25 mg/kg treatment groups remained consistently lower than PRG (< 30–50%) and SG soils (< 23–71%) over the 28-day experimental period (Fig. 1).

Plant uptake of Se occurred in a dose-dependent manner across all grass species (Fig. 1). There was a significant difference between Se uptake across plant species, with PRG plants accumulating 280% more Se than either Bahia or SG tissues in both the 15 and 25 mg/kg treatment groups over a 24-h period (Fig. 1 and Table 1). Within a given species, concentrations of plant-associated Se did not significantly change over time even as soil concentrations steadily decreased (Fig. 1, Table S1). This suggests that the decrease in Se in the soil fraction was likely due to water loss and desorption rather than loss due to plant uptake. The increase of Se in plant tissue can be attributed to grasses’ ability to accumulate and appropriate Se in low concentrations, leading them to be good candidates for phytostabilization (Schiavon & Pilon‐Smits,^2017^). However, the plateau of Se concentrations can be explained by the fact that at high heavy metal exposure, plants can experience nutrient starvation through the disruption of nutrient uptake and enzymatic activity (2025). While Jiang et al., 2022; Umar et al., 2025 the primary focus of this study is to identify grass species that can display a clear dose–response to find a correlated signature reflectance for each treatment level, our preliminary results suggest that PRG grasses are effective at phytostabilizing both metals. Further studies are required to investigate the phytoremediation potential of the three grasses.

Arsenic speciation was performed at the end of the experiments in plant tissue to determine the form of As stored. The GFAAS analysis separated organic species monomethylarsonic acid (MMA), dimethylarsinic acid (DMA), and unretained organic species tentatively labeled as arsenobetaine (AsB), as well as the inorganic species As(III) and As(V). Inorganic As species such as As(V) and As(III) are readily phytoavailable, with As(V) being considerably less toxic than As(III) and DMA, in that order (Meharg & Hartley‐Whitaker, 2002). Arsenic speciation showed that inorganic As was the major As species present in the plant tissue, with As(V), the spiked form of As, representing the majority of inorganic species (Fig. 2). Relatively small amounts of the organic species DMA and AsB were present at all dosage treatments across all plant species. There is no known mechanism of plant transformation of As(V) into organic As species (Zhao et al., 2009), indicating that the remaining As species reflect the concentrations of pre-existing As in the soil. No MMA was detected across all the samples (data not shown).

### Spectroradiometer analysis

**Fig. 3:**
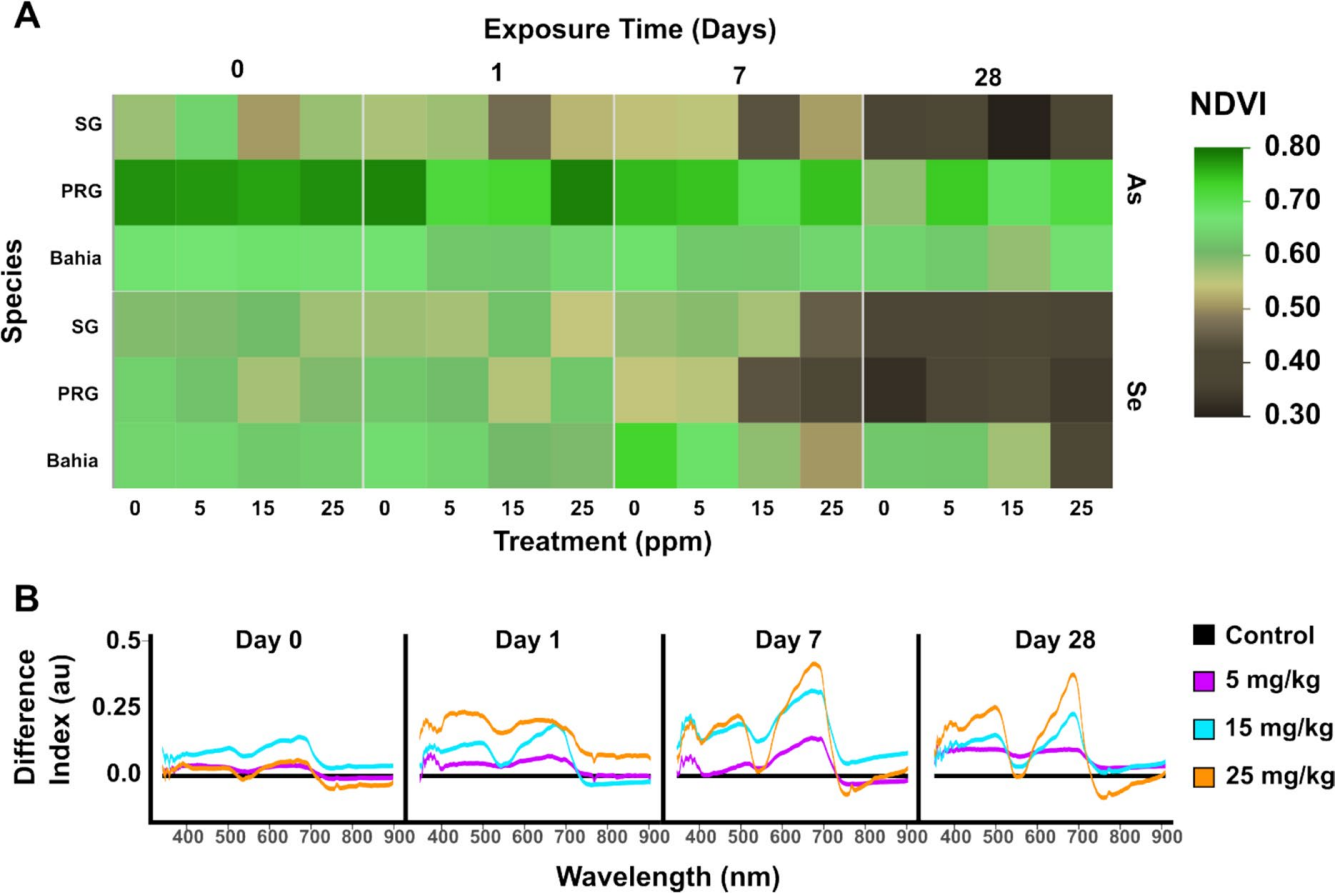
Analysis of SVC collection showing the resulting **A** average NDVI of three replicate collections across all samples where lower values (brown) represent unhealthy or dead plants and higher values (green) represent healthy plants and **B** average spectra of Bahia treated with Se and showing a clear dose response across the full range of treatments. SVC response is normalized to the control

Spectroradiometer (SVC) reflectance can be used to track plant activity over a broad range of wavelengths—encompassing the near infrared (NIR), visible, and near ultraviolet (NUV) (Figure S5). While SVCs do not capture data in a spatially resolved manner, it is a useful tool to quickly capture the average spectra from the field of view of the aperture. The normalized difference vegetation index (NDVI) is a broadly used vegetation index that calculates plant health using the NIR and red spectrum, indicative of the relative water content and chlorophyll concentration, respectively (Evangelides & Nobajas, 2020; Lassalle et al., 2020; Pettorelli et al., 2005). While other indices were tested using our dataset, the NDVI was the only index that consistently differentiated between healthy and dead plant material (Figure S4). The NDVI scale is –1 to 1, with values between –1 and 0 representing dead plants on inorganic material, unhealthy plants represented between 0 and 0.4 and values from 0.4 to 1 representing increasingly healthy plants. In the greenhouse study, NDVI values never exceeded 0.8 or fell below 0.3, although plants visually appeared healthy at the start of the experiment and, in some cases, were clearly dead by the end, validated by visual monitoring. A heatmap of the calculated NDVI values shows the degree to which plant health changed over the timeframe of the experiment according to plant species, metal treatment, and dose (Fig. 3A).

There was no statistical correlation between As treatment dose and NDVI response for any of the grass species (Fig. 3A). SG was the only plant to decline in health over the various collections, but still did not provide a response to treatments. In contrast, all Se treated grasses showed a decrease in NDVI as a function of increased Se treatment concentrations from days 0, 1, and 7 (Fig. 3A). A week post treatment, Bahia displayed a clear response to the Se treatment, as seen with NDVI values declining with increasing concentration applied. At day 28, the SG and PRG plants exhibited low NDVI values independent of Se treatment dose, whereas Bahia continued to show a dose-dependent response to Se. This decline in SG health during the Se treatment study was likely due to high temperatures in the greenhouse since control plants declined in health. The average spectra of Bahia clearly show a dose response as early as 24 h post Se exposure, showing the reflectance deviating from control plants based on treatment—with this deviation from control becoming increasingly clear over the timeframe of the experiment (Fig. 3B). PRG plants exhibited a significant difference in response between the control/low concentration treatments and high concentration treatments (Table S1), meaning that there was a significantly different response between plants past the 5 mg/kg threshold. This is confirmed with previous Se toxicity studies where above 5 mg/kg dry weight results in plant health decline and withering (Szőllősi et al., 2022).

The differences in plant responses to As and Se can be explained by the role each element plays in plant development. At high levels of Se, selenoprotein structures are damaged as the amino acid cysteine is replaced by selenocysteine (Ali et al., 2021; Gupta & Gupta, 2017). Se toxicity can result in stunted root and plant growth, as well as induce the production of reactive oxygen species (ROS) (Somagattu et al., 2024). ROS cause damage to cell membranes and result in cell damage and oxidative stress due to an excess of hydroxyl radicals (Choudhury et al., 2017). However, at low concentrations, Se is an essential component of plant defense against abiotic stressors, as seen with the selenoprotein glutathione peroxidase, which reduces hydrogen peroxide to prevent damage to cellular machinery (Madhu et al., 2023). Additionally, at low concentrations, Se has been found to improve photosynthetic rates, nutrient uptake, and growth (Ali et al., 2021; Gupta & Gupta, 2017).

As is not essential for plant health and can cause a wide variety of detrimental effects depending on its speciation (Abbas et al., 2018). One of the effects of As is the overproduction of hydrogen peroxide resulting in lipid peroxidation (Abbas et al., 2018; Rafiq et al., 2017). Due to the overall deleterious nature of As in plant systems, it can explain why the three grass species had an increased adverse response to As compared to Se. Additional trace elements of CCR such as cadmium, chromium, nickel, lead, antimony, thallium, and zinc could be used to further our understanding of how individual elements impact plant systems within the broader CCR context (Senior et al., 2020).

The selection of bioindicators for a particular region must reflect the plant’s resistance to seasonal changes as well as their carbon metabolism. The three grasses have a diverse range of preferred air temperatures: 24–29 °C for SG, 20–25 °C for PRG, and 27–32 °C for Bahia (Gibeault et al., 1972; Lemus, 2022; Renz et al., 2009). A week before the last collection (28 days post treatment) for Se groups, the temperature inside the greenhouse reached over 30 °C, which is above the air temperature range for SG and PRG. Plants with C4 metabolism have been known to have reduced photosynthetic rates and stunted growth (Zhang et al., 2025). This can explain why SG and Bahia were more impacted than PRG by heat stress, since PRG grass has a C3 metabolism, whereas the others have C4. Additionally, plants undergoing heat stress experience a large release of ROS that may damage plant tissue, leading to senescence (Bhattacharjee, 2005). Due to the confounding effects of metal toxicity with the heat stress, it is difficult to draw conclusions about the driving factors impacting the declining plant health. However, it is possible that the compounding effect of already elevated ROS levels in plant tissue due to the introduction of contaminants, as well as growing outside the preferred temperature range, could have caused SG to die at day 28.

Spectra collected with the SVC are not spatially resolved and thus include background spectra from the soil and other non-grass materials, such as plant trays, that may cross into the field of view of the collection window. Despite these qualifications, the resulting NDVI calculated from the collected SVC spectra reflects the observed changes in plant health over time, indicating that this method is sufficient for a quick capture of plant health. The plant’s NDVI response did not distinguish between plants stressed due to heat or pathogen pressure.

Comparing the chemical analysis with NDVI data, we can conclude that some grass species are better bioindicators of select stress than others. Selenium treatment produced notable plant responses within treatment, while As treatment resulted in gradual plant decline over time. While the chemical analysis confirms a dose response assimilation of contaminants in all grass species, not all grass species displayed an NDVI dose response. This could be due to an increase in activation for antioxidant enzymes to resist the impacts of heavy metal accumulation by removing ROS, thereby mitigating the detrimental effects on cell organelles without removing the heavy metal species themselves (Jiang et al., 2022). PRG and SG species have been known to respond to Se via phytoextraction by accumulating it in harvestable parts such as shoots, whereas Bahia lacked any notable Se and As tolerance mechanism (Matichenkov, 2017). Thus, for a plant to be considered a good bioindicator, the plant species and mechanism response to heavy metals must be considered.

The pot experiment has revealed that dose response differs with grass species and that all grass species were more sensitive to Se treatments. Both Bahia and PRG were highlighted as the best candidates for further study as bioindicators, as they both displayed relatively clear dose responses to Se and were generally more hardy plants than the SG species. For field-scale studies, we chose to use PRG, as this species is more cold-hardy than Bahia and the field-scale study was planned during colder autumn months (Keep et al., 2021).

### Field greenhouse NDVI analysis

**Fig. 4:**
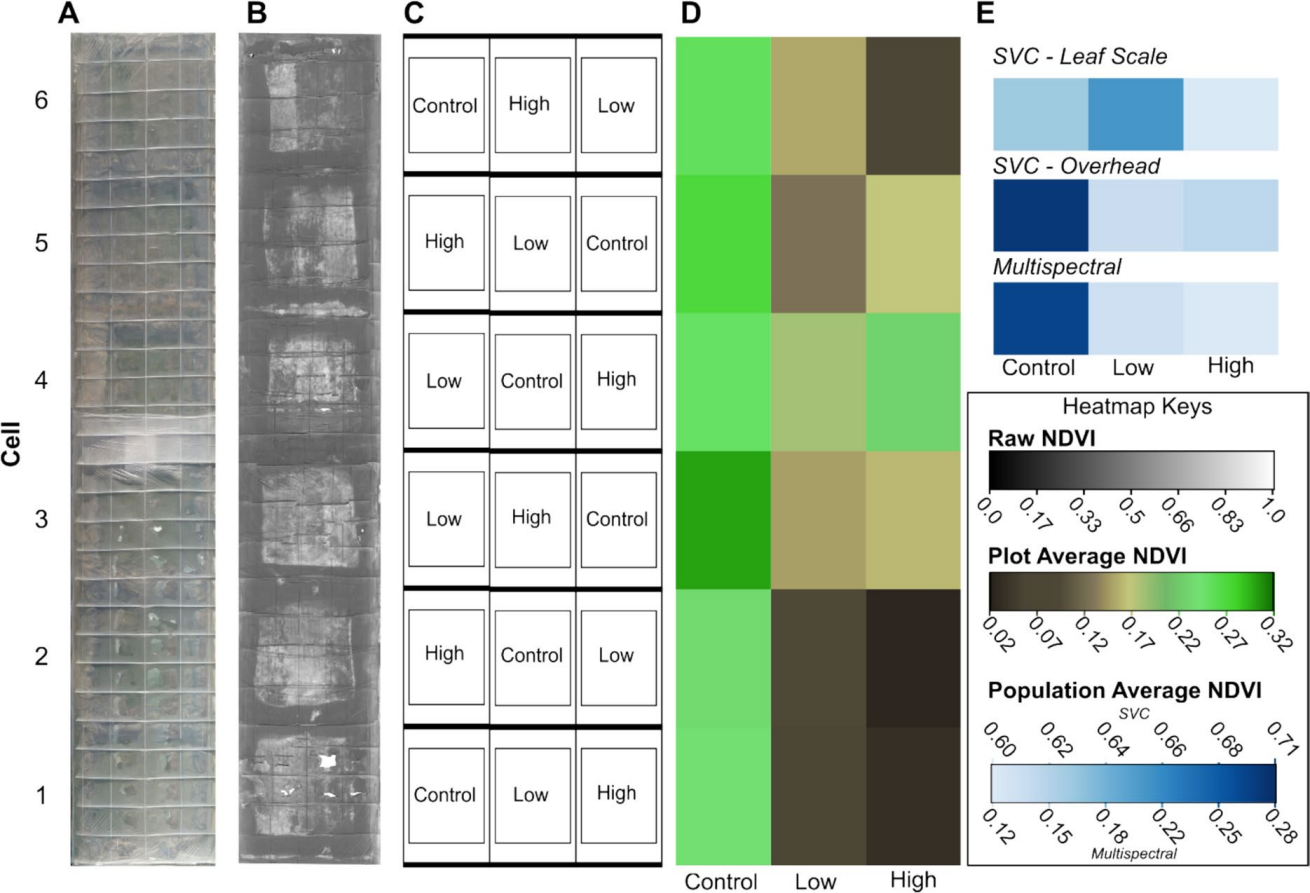
Field experiment assessment 78 days post seeding. **A** Orthorectified UAV image over the length of the field greenhouse. **B** Raw NDVI values overlayed onto the orthorectified UAV image. **C** Randomized treatment layout of each field plot. **D** Mean NDVI of all pixels in a given treatment for each field plot rearranged from left to right with increasing CCR treatment concentration. **E** Average NDVI per treatment across all plots measured by both SVC and multispectral cameras. Each cell was 2 m by 6.1 m

UAV mounted multispectral sensor collections were used to calculate an average NDVI score for each pixel for the entire field spectral collection (Fig. 4). The NDVI calculated from the multispectral sensor confirmed that cells with low and high concentrations of CCR have NDVI lower values, indicating that the grasses were less healthy compared to the control groups (Fig. 4D). Control sections were found to be statistically different from both the low concentration sections (*t*-test: *p* = 1.62E−4) and high concentration sections (*t*-test; *p* = 6.80E−3). For cells 1–3, the condensation below the plastic film of the greenhouse resulted in patches of high reflectance. Cells 4 and 5 also displayed an increase in plant health as the concentration increased from low to high. The variability in response can be due to the composition of the CCR, which is a blend of several heavy metals including lead and chromium. Due to the heterogeneous content of the CCR applied to each section, the lack of consistency in NDVI results may be due to an uneven mix of the contaminant per cell. Cells 3 and 4 (Fig. 4A) were located on either side of the overlapping plastic sheet used for the roofing. The overlap produced a noticeable shadow, which may have contributed to the plant health of the two cells. It is possible that the plastic overlap decreased light intake for the plants, hindering their growth.

The average NDVI calculations for control, low, and high sections varied for SVC and multispectral collections and are shown in Fig. 4E. Leaf scale depicts low concentrations as healthier plants than control and high concentration groups, contradicting the analysis completed on the multispectral collections. Overhead SVC data show plants being healthy at control, declining in health at low concentration, and improving at higher concentrations. Aerial multispectral collections showed a decrease in plant health with increasing CCR concentrations in the soil. Leaf scale is more accurate in assessing the health of the individual plant by avoiding analyzing bare soil in the background and can capture the vertical profile of CCR distribution within the grasses (Arellano et al., 2017). Leaf scale, however, fails to capture the variability that might occur within the sampled cell sections, potentially leading to mischaracterization of the plant response. Overhead SVC followed the multispectral collections more closely, which could be due to the capturing of background bare soil that lowers NDVI values. This inverse relationship between bare soil and NDVI can also result in overestimation of the impacts of stress caused by CCR (Lassalle et al., 2020). 

From the analysis of CCR impacts on plant health, multispectral imaging is the most effective method in determining a dose response in PRG grasses due to its ability to use the aerial vantage point and capture the entire field greenhouse. When considering large field monitoring, it is also the most feasible option as it avoids on-site scanning of individual plants or taking samples for extensive chemical testing. However, in a smaller and controlled environment, the SVC data collections were a reliable method of exploring NDVI trends.

Limitations of both field spectrometers and UAV-mounted imaging spectrometers include potentially high upfront instrument purchase costs, instrument maintenance (e.g., calibration) expenses, and required personnel expertise to collect high-quality measurements and interpret the data. The spectroradiometer market is projected to grow at a compound annual growth rate of 7.1% from 2022 to 2027, which has incentivized the industry to diversify their models to accommodate a wider range of budgets (Verified Market Reports, 2025). Similarly, the cost of hyperspectrometers and hyperspectral cameras has declined over the years. However, their performance continues to be constrained by environmental conditions during data acquisition (Podlesnykh et al., 2024). UAV flights for this experiment were limited to days and times when there was minimal cloud cover, stable atmospheric conditions, and no condensation or snow on top of the greenhouse roof. There was an attempt to minimize the impact of shadows by timing the flight to be when the sun was directly above (nadir) the greenhouse. In areas with frequent inclement weather, regular UAV observances may therefore be more challenging to capture.

**Fig. 5:**
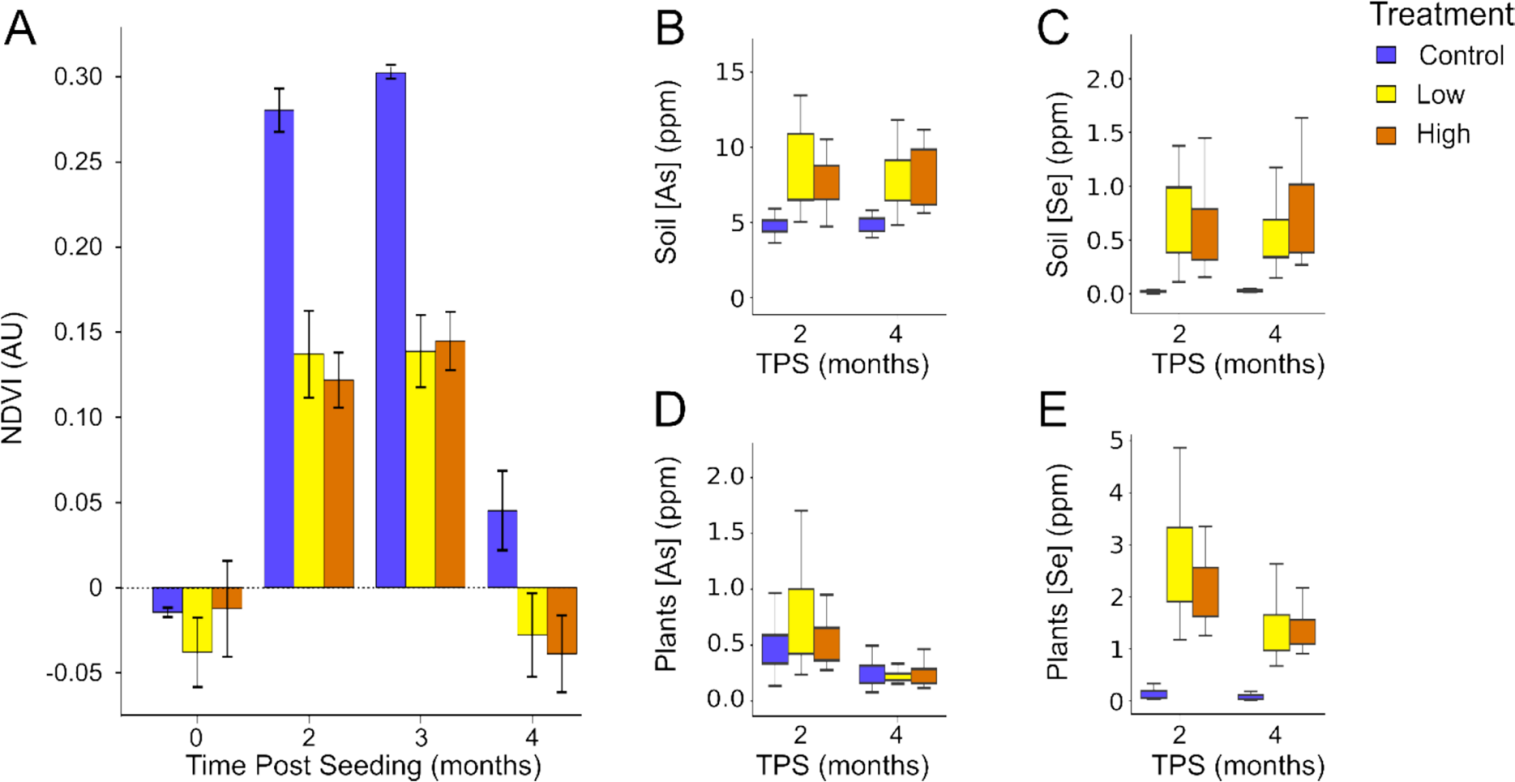
PRG responses to CCR treatment over time by treatment condition. Data represent all 6 test plots as biological replicates. Average NDVI collected via multispectral drone. Error bars represent standard error (**A**). Soil concentration of As (**B**) and Se (**C**) at 2 and 4 months’ time post seeding (TPS). Above ground plant tissue concentrations of As (**D**) and Se (**E**) at 2 and 4 months TPS

The seasonal and CCR intensity impacts on grass health were explored as a function of time post seeding (TPS), sampling over the course of four months (Figure S6). Cells with the same treatments were binned together (Fig. 5A). As time progressed, control plots went from being barren (0 TPS) to being covered with grass (2–3 TPS) and finally declining in plant health until the vegetation died at 4 TPS. Although these plants were grown in a greenhouse and shielded from harsh environmental swings, the field greenhouse was not heated and 4 TPS was collected during December, leading the plants to go dormant due to freezing temperatures. The NDVI averages were different between collections (one-way ANOVA, *p*-value = 9.35e−33) and significance was confirmed by a Tukey’s HSD test. Results show that 2 and 4 TPS were significantly different from 0 TPS, and there was no significant difference between 0 TPS and 4 TPS (Table S2). This demonstrates that seasons significantly impact average NDVI, regardless of treatment.

Further analysis, binning data by treatment, shows that both the low and high treatments were significantly different from control groups, while not significantly different from each other (Table S3). This indicates that there is a positive correlation between the spectral response of PRG and elevated CCR in the soils, but there is no indication that it can differentiate between low and high concentrations. Further studies are needed to examine PRG sensitivity to varying CCR applications.

Results from chemical analysis of soil samples is depicted in Fig. 5B-–C for each TPS. Kruskal–Wallis and Tukey’s post hoc test indicate there was no significant difference between the soil concentration profiles for As and Se across TPS and treatment types (Table S4). Chemical analysis for plant tissue revealed no significant difference between low and high treatments but significantly different from the control groups (Fig. 5D–E and Table S5). Interestingly, while there is a significantly higher concentration of As in the treated plots from control, plant tissues are not significantly different at either two or four months TPS.

There was no analysis done on below-ground biomass; therefore, the accumulation of heavy metals in roots during the transfer process of roots-to-shoots was not accounted for. The decrease in metal concentration in plant tissue between the 2 months of sampling coincides with the decrease in NDVI (Fig. 5). This observation, which Se and As decreased in the above-ground plant tissue between months 2 and 4, may be explained by the plants undergoing senescence. Plants undergoing senescence have been shown to increase metal mobilization from dying leaves to roots as a form of nutrient storage (Maillard et al., 2015). While the concentration differences between the collection times are insignificant, further studies need to be conducted to determine how transfer factors might change with seasonality by calculating the metal concentration in roots (Masotla et al., 2023). Discrepancy between As and Se scales for plant tissue could be explained by Ca, Mg, and P-rich soils decreasing As (Li et al., 2010a, 2010b). The soils used in the field study were locally sourced and are representative of the calcareous soils typical of New England, which originate from the region’s underlying limestone bedrock (Beaumont, 1942).

Results from this investigation suggest it is advisable for CCR impoundments to monitor CCR distribution and degradation through aerial imaging given that appropriate grass species are used. Expanding the application of UAV-based heavy metal detection in soils could be particularly valuable for monitoring contamination at military installations and firing ranges, which are often impacted by elevated concentrations of trace metals (Broomandi et al., 2020; Choi et al., 2011; Clausen & Korte, 2009). Bahia and PRG grasses have shown to be good bioindicator candidates in certain contexts, with factors such as temperature and contamination types influencing plant response. Further studies should be conducted over a prolonged period to determine the maximum contaminant loading concentration in plants, as well as plant-soil transfer mechanisms for other contaminants. It is estimated that between 2000 and 2020, 2.4 trillion tons of CCR were produced, 1.1 trillion reused, and 1.1 trillion disposed (Deonarine et al., 2023). CCR disposal sites are especially vulnerable to intensive rainfalls, flooding, or natural disasters which could cause CCR to be introduced into surface waters and groundwater (Foulds et al., 2014). In 2019, 91% of US coal-fired power plants with regulated landfills have been found to have unsafe levels of ash components in downgradient wells (Petrović & Fiket, 2022). Current monitoring practices are centered around rigorous surface water sampling campaigns and computing water quality index to ensure heavy metal pollution meets compliance standards (Petrović & Fiket, 2022; Ruhl et al., 2012). The most prominent tool for CCR assessment is US EPA’s Leaching Environmental Assessment Framework (LEAF) that estimates leachability under various environmental and site disposal conditions (Da Silva et al., 2018). LEAF methods rely on leachate samples in order to calculate innate sample qualities such as pH, liquid-to-solid ratios, and a slew of EPA laboratory methods which can be time-consuming and laborious for active monitoring (Thorneloe et al., 2017). UAV fly-overs and data analysis would be a less time and resource-intensive process than completing a series of chemical tests. Further steps in bioindicators research should focus on developing novel unintrusive frameworks which can track CCR fate in protected waters to predict accumulation sites for monitoring and risk assessments.

## Conclusion

This study explored the biomechanical response of plants to the addition of Se and As into soils. Metal transfer analysis showed that PRG plants accumulated five times more Se than either Bahia or SG tissues over a 24-h period. Additionally, As speciation analysis showed that As(V) had the highest concentration in plant tissue. Spectroradiometer analysis for the pot studies showed the different stress responses to metal concentrations and increasing air temperatures. SG showed declining health on the 28th day of sampling for As due to the heat stress. PRG was chosen to be used in the field greenhouse study due to its resilience to the cold and its ability to display a dose–response to the four differing treatments in the Se trials. Three different monitoring technologies (SVC at leaf scale, SVC overhead, and multispectral imaging) were tested in the field experiment. The study reveals that the multispectral camera was able to detect healthy plants in the control plots and plants declining in health as the CCR treatment increased. SVC at leaf scale was unable to capture variability in plant health in a plot, whereas SVC overhead was not sensitive at distinguishing bare ground from dying plants. Effects of seasonality and CCR treatments were explored further in the field experiment. Both seasons and CCR treatments significantly impact the average NDVI of PRG grasses; however, PRG lacks the sensitivity to produce a signature NDVI value for each treatment. The results suggest that further studies are needed to determine whether increasing CCR in soils will also increase the metal mobilization to roots during plant senescence. The use of hyperspectral imaging for biomonitoring purposes is promising; however, additional research is also needed to establish the relationship between chemical contaminants and environmentally relevant biomonitoring plants. As we have shown in our study, not all grass species are appropriate for biomonitoring, and Bahia and PRG grasses are not likely to be suited for growth in all environments where biomonitoring is required.

## Supplementary Information

Below is the link to the electronic supplementary material.

ESM 1 (DOCX 2.17 MB)

## Data Availability

Data is available by request to corresponding author.
