# Supplementary material for: Grass is a tattletale: using grass as a biomonitoring tool for remote sensing of coal combustion residue contamination
Source: Environ Monit Assess. 2025 Dec 9;198(1):29. doi: 10.1007/s10661-025-14719-7 (PMC12689824; doi:10.1007/s10661-025-14719-7)
Supplement: Supplementary file 1 — (DOCX 2.17 MB) [file 10661_2025_14719_MOESM1_ESM.docx]

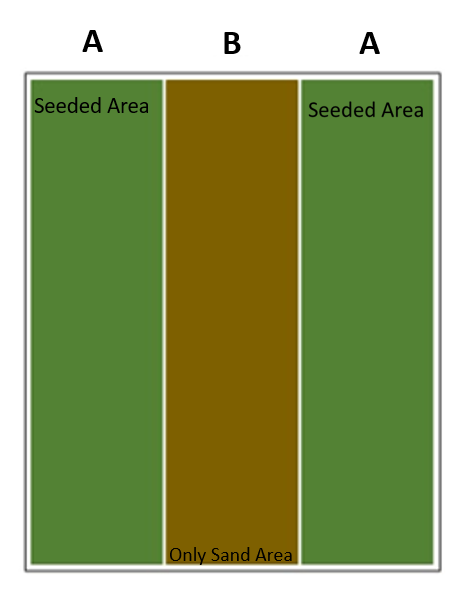


Figure S1: Displays double mesh tray blocking pattern design for plant seeding. Sections ‘A’ are the only areas where seed was applied. Section ‘B’ is an area where only sand was in the tray, and nothing was seeded so that the different levels of ppm of As or Se could be applied.


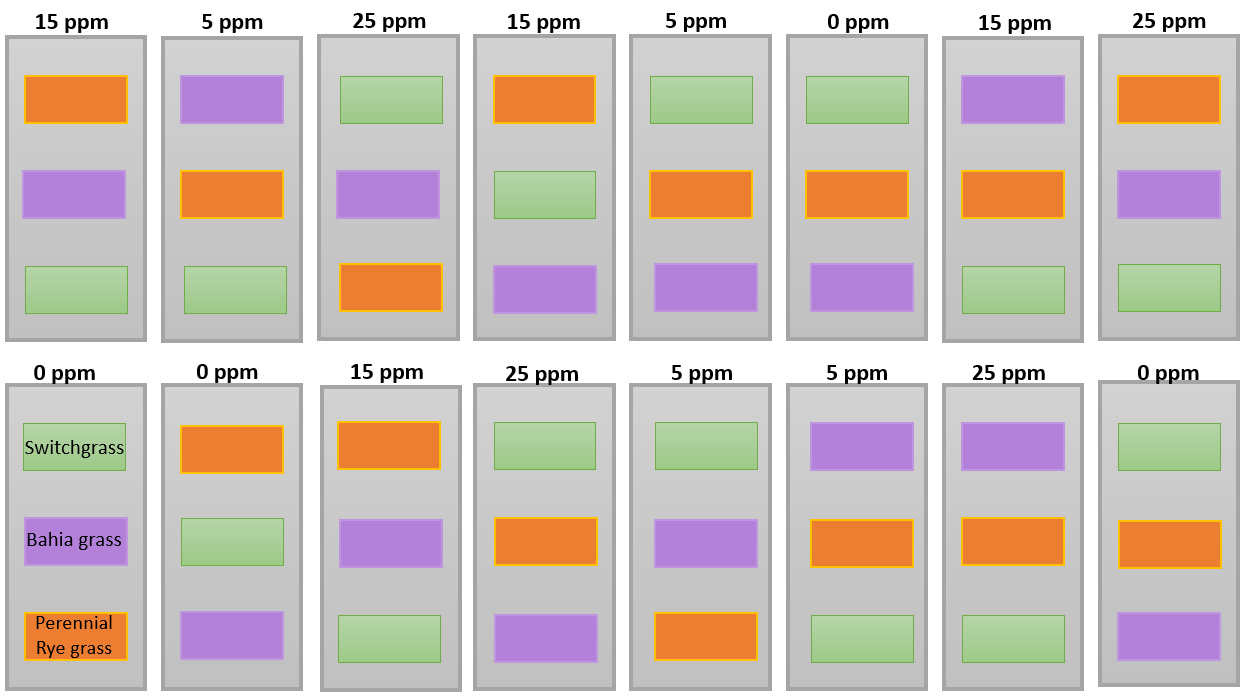


Figure S2: Displays experiment design randomization and blocking of three grass species within one containment tray. Containment trays were separated by the varying levels of ppm of As or Se depending on the experiment. Each varying level of ppm was replicated four times. Grey boxes represent containment trays. Green boxes represent double mesh trays with switchgrass (SG) planted. Purple boxes represent double mesh trays with Argentine Bahia Grass (Bahia) planted. Orange boxes represent double mesh trays with Perennial Rye Grass (PRG) planted.

Table S1

| Sample Name |  | Be | Al | V | Cr | Mn | Fe | Co | Ni | Cu | Zn | As | Se | Sr | Mo | Ag | Cd | Sn | Sb | Ba | Hg | Tl | Pb | U |
| --- | --- | --- | --- | --- | --- | --- | --- | --- | --- | --- | --- | --- | --- | --- | --- | --- | --- | --- | --- | --- | --- | --- | --- | --- |
|  |  | mg/kg | mg/kg | mg/kg | mg/kg | mg/kg | mg/kg | mg/kg | mg/kg | mg/kg | mg/kg | mg/kg | mg/kg | mg/kg | mg/kg | mg/kg | mg/kg | mg/kg | mg/kg | mg/kg | mg/kg | mg/kg | mg/kg | mg/kg |
| Drum 1 |  | 3.13 | 26570.29 | 153.55 | 63.09 | 88.66 | 43025.84 | 10.77 | 28.64 | 28.92 | 48.96 | 91.06 | 19.26 | 439.68 | 10.88 | 0.04 | 0.66 | 4.10 | 1.23 | 212.17 | 0.11 | 1.09 | 15.38 | 3.43 |
| Drum 2 |  | 2.97 | 25810.47 | 148.78 | 61.60 | 87.81 | 41328.93 | 10.48 | 27.48 | 28.61 | 48.42 | 93.36 | 19.09 | 429.02 | 11.24 | 0.04 | 0.65 | 4.27 | 1.37 | 207.91 | 0.15 | 1.19 | 15.23 | 3.38 |
| Drum 3 |  | 3.18 | 26336.78 | 156.71 | 67.10 | 93.46 | 54305.98 | 12.07 | 31.41 | 29.91 | 62.62 | 94.72 | 19.08 | 432.16 | 12.58 | 0.05 | 0.66 | 4.78 | 1.77 | 207.62 | 0.20 | 1.16 | 15.80 | 3.47 |
| Drum 4 |  | 3.70 | 26217.64 | 151.16 | 63.08 | 89.09 | 44749.37 | 10.97 | 28.61 | 28.68 | 48.20 | 90.22 | 19.42 | 453.85 | 11.88 | 0.04 | 0.64 | 4.40 | 1.26 | 213.66 | 0.24 | 1.23 | 15.62 | 3.50 |
| Drum 5 |  | 3.40 | 24827.42 | 147.31 | 60.87 | 86.88 | 43786.64 | 10.66 | 27.54 | 27.48 | 46.34 | 87.68 | 18.77 | 437.09 | 11.73 | 0.04 | 0.66 | 4.43 | 1.42 | 205.34 | 0.31 | 1.19 | 15.28 | 3.45 |
| Drum 6 |  | 2.98 | 24119.06 | 143.31 | 58.30 | 84.96 | 40557.18 | 10.11 | 26.33 | 26.78 | 45.31 | 84.72 | 18.79 | 423.48 | 11.20 | 0.04 | 0.64 | 4.26 | 1.41 | 200.71 | 0.30 | 1.28 | 14.81 | 3.39 |
|  |  |  |  |  |  |  |  |  |  |  |  |  |  |  |  |  |  |  |  |  |  |  |  |  |
| Quality control |  |  |  |  |  |  |  |  |  |  |  |  |  |  |  |  |  |  |  |  |  |  |  |  |
| Calibration check | % recovery | 109% | 137% | 117% | 107% | 114% | 117% | 114% | 111% | 106% | 111% | 113% | 121% | 121% | 139% | 100% | 104% | 158% | 113% | 112% | 45% | 89% | 94% | 90% |
| SRM NIST 2711 | %recovery |  | 124% | 128% | 112% | 114% | 115% | 108% | 111% | 107% | 109% | 126% |  | 110% |  | 116% | 102% |  |  | 112% |  |  | 104% |  |
| Duplicate drum 1 | % difference | 10% | 6% | 3% | 2% | 1% | 6% | 1% | 0% | 2% | 2% | 3% | 5% | 3% | 8% | 6% | 4% | 6% | 31% | 1% | 85% | 22% | 2% | 3% |

Table S1 shows the ICP-MS measured concentrations of each heavy metal detected in the coal combustion residue applied to the field study containment cells 1-6. All 6 drums were mixed together to get an average composite mixture from each for the treatments.


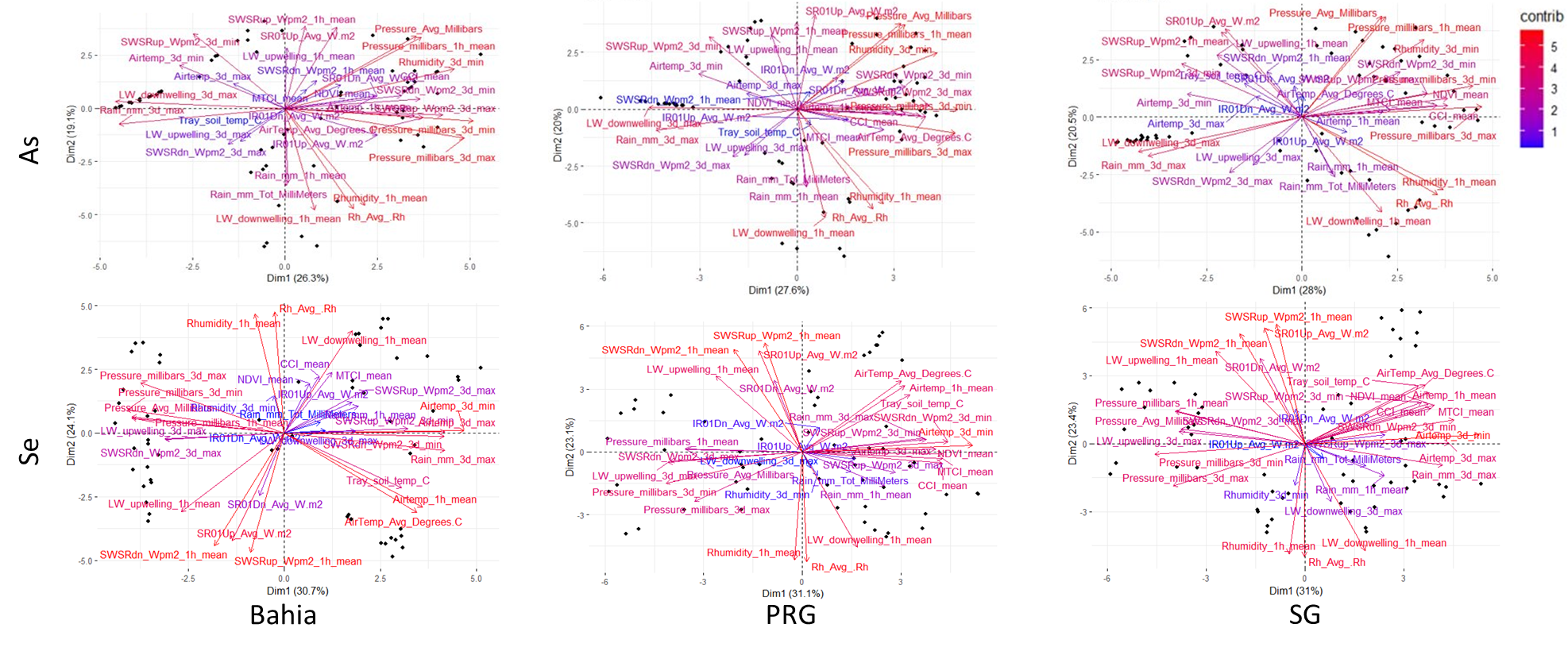
Figure 3S. As and Se PCA biplots including meteorological parameters. As had the lowest correlation with weather parameters than Se, however both yielded low correlations, suggesting that weather attributes were not a major influence on plant health in the greenhouse.


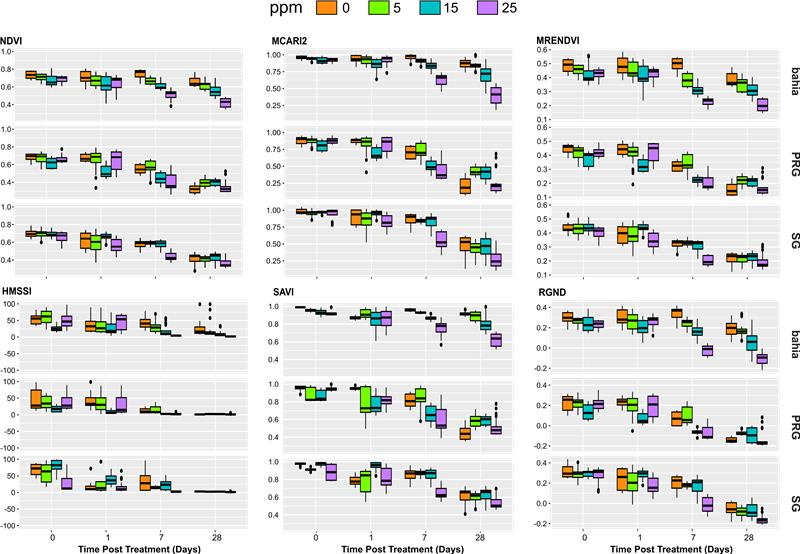


Figure S4. Analysis of different vegetative indices to find the best metric for plant health. Normalized Difference Vegetation Index (NDVI) is the calculation of the difference in NIR reflectance and Red reflectance over the sum of the two parameters. NDVI can be used to assess fraction of absorbed photosynthetic active radiation of the plants analyzed (Pettorelli et al, 2005). Modified Chlorophyl Absorption Ratio Index (MCARI2) is one of several CARI indices that indicates the relative abundance of chlorophyll. This modified version of MCARI is considered a better predictor of leaf area index (Daughtry et al, 2000). Modified Red Edge Normalized Difference Vegetation Index (MRENDVI) emphasizes the vegetation red edge and is more sensitive for observing plant regeneration and growth of understory plants obscured by tree canopies (Evangelides et al, 2020). Heavy Metal Stress Sensitivity Index (HMSSI) uses reflectance values at particular wavelengths for heavy metal detection (Zhang et al, 2018). Soil Adjusted Vegetation Index (SAVI) minimizes soil brightness and highlights vegetation using red and NIR wavelengths (Huete, 1988). Red-Green Normalized Difference Index (RGNDI) is a custom function created by authors to find the contrast between background spectra and vegetation. NDVI was chosen over the other indices because NDVI means decreased with time and concentration, creating a clear divergence between the control groups and the treatment groups.


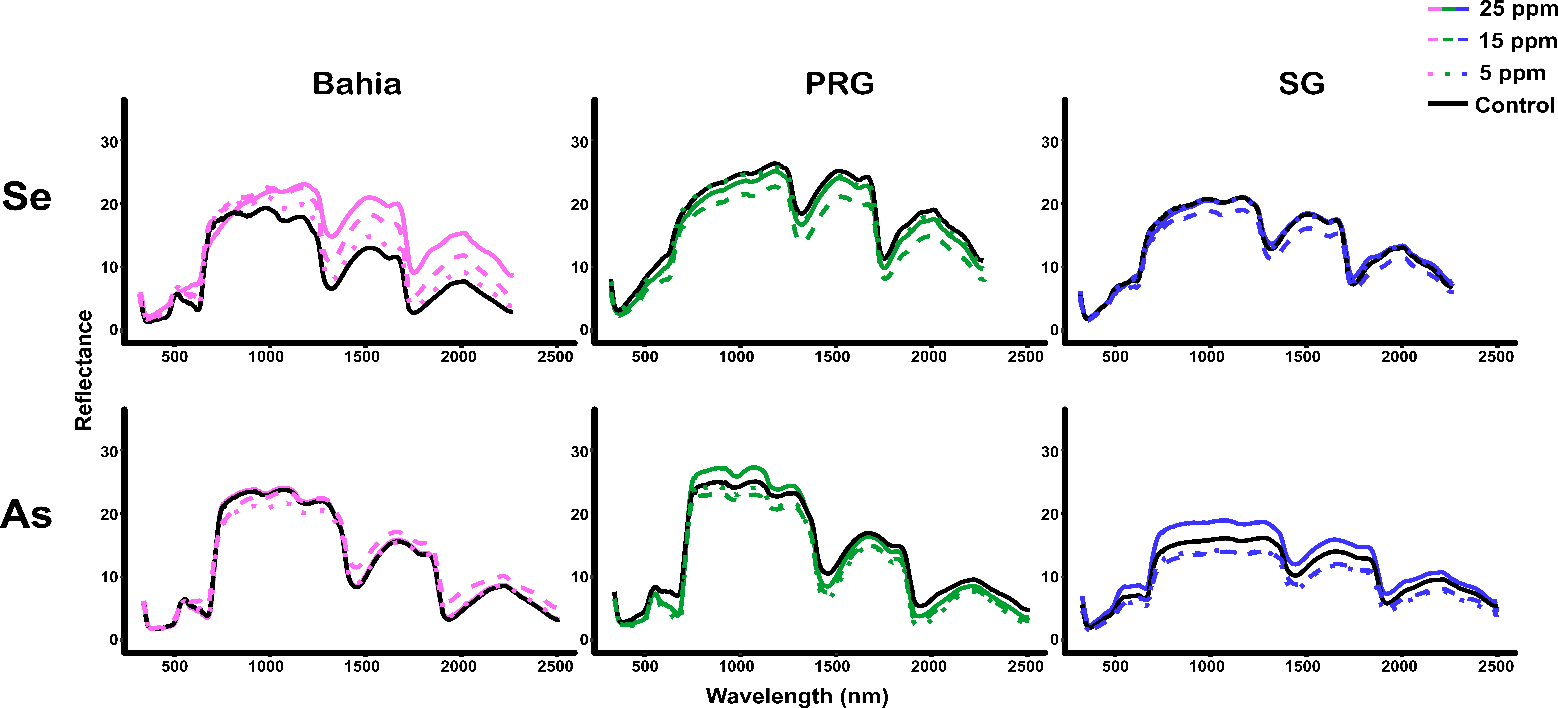


Figure S5. Spectroradiometer reflectance of all plants after 28 days of exposure to Se or As. Values for each treatment represent an average of 4 replicates. At the end of the experimental timeline, Bahia grasses exhibited a directional dose response to Se in both the 600nm and 1000-2200nm regions. Increased reflectance in the 600nm region is indicative of a stress response at the scale of photosynthetic pigments such as chlorophyll while increases above 1500 can indicate water stress. While the Bahia plants exposed to Se exhibited reflectance signatures consistent with toxin exposure, the As exposed Bahia did not. The PRG and SG species did not display an observable hyperspectral response when exposed to Se, and only at the highest dose As exposure was there evidence of heavy metal stress on SG. Taken together, these data indicate different grass species have different tolerances and hyperspectral response signatures to heavy metal exposure.


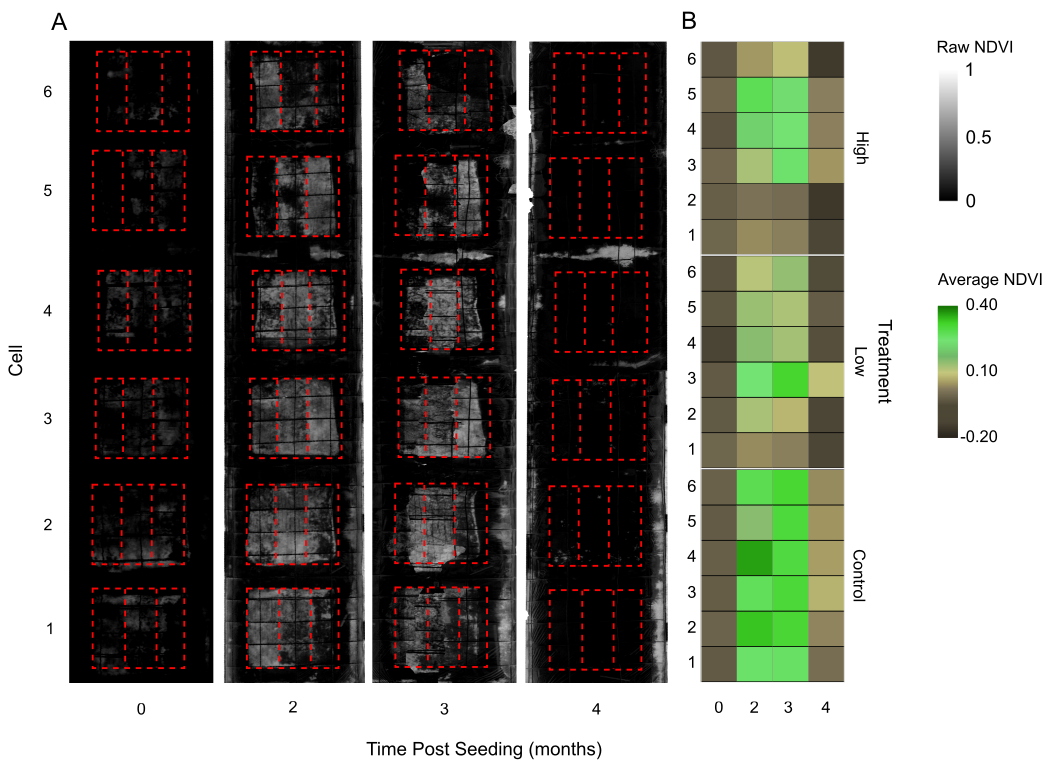

Figure SX:

Table S2: Tukey’s HSD p-values for Temporal Analysis of NDVI

|  |  | Time Post Seeding (Months) | | | |
| --- | --- | --- | --- | --- | --- |
|  |  | 0 | 2 | 3 | 4 |
| Time Post Seeding (Months) | 0 | 1 | 0 | 0 | 0.86071 |
|  | 2 | 0 | 1 | 0.848684 | 0 |
|  | 3 | 0 | 0.848684 | 1 | 0 |
|  | 4 | 0.86071 | 0 | 0 | 1 |

Initial ANOVA’s test resulted in p-value= 9.350 E-33

S3: Tukey’s HSD p-values for Concentration Analysis of NDVI

|  |  | Concentration of CCR Applied | | |
| --- | --- | --- | --- | --- |
|  |  | Control | Low | High |
| Concentration of CCR Applied | Control | 1 | 3.10E-05 | 5.51E-05 |
|  | Low | 3.10E-05 | 1 | 0.992155 |
|  | High | 5.51E-05 | 0.992155 | 1 |

Initial ANOVA’s test resulted in p-value= 5.9045E-5

| **Table S4: Tukey's HSD Pairwise Group Comparisons Test for As and Se Concentration in Soil Samples.** Groups were defined by their time post seeding (2 and 4) and treatment level (control, low, high). Kruskal-Wallis test was first completed and showed significant difference in As (p-value=1.28E-9) and Se (p-value=3.49E-17) concentration profile. (*) signifies that there was a significant difference between the groups (p-value>0.05 | | | | | | |
| --- | --- | --- | --- | --- | --- | --- |
| **As** | | | | | | |
|  | Control 2 | Control 4 | High 2 | High 4 | Low 2 | Low 4 |
| Control 2 | 1.00E+00 | 7.73E-01 | 8.49E-01 | 6.13E-01 | 1.95E-01 | 7.16E-01 |
| Control 4 | 7.73E-01 | 1.00E+00 | 1.36E-01 | 1.00E+00 | 5.52E-03 | 1.00E+00 |
| High 2 | 8.49E-01 | 1.36E-01 | 1.00E+00 | 6.82E-02 | 8.60E-01 | 1.09E-01 |
| High 4 | 6.13E-01 | 1.00E+00 | 6.82E-02 | 1.00E+00 | 1.85E-03* | 1.00E+00 |
| Low 2 | 1.95E-01 | 5.52E-03 | 8.60E-01 | 1.85E-03* | 1.00E+00 | 3.99E-03* |
| Low 4 | 7.16E-01 | 1.00E+00 | 1.09E-01 | 1.00E+00 | 3.99E-03* | 1.00E+00 |
| **Se** | | | | | | |
|  | Control 2 | Control 4 | High 2 | High 4 | Low 2 | Low 4 |
| Control 2 | 1.00E+00 | 1.00E+00 | 1.52E-14* | 5.00E-08* | 1.52E-14 * | 3.86E-07 * |
| Control 4 | 1.00E+00 | 1.00E+00 | 1.52E-14 * | 1.24E-08 * | 1.52E-14 * | 1.03E-07 * |
| High 2 | 1.52E-14 * | 1.52E-14 * | 1.00E+00 | 3.33E-04 * | 2.13E-01 | 1.76E-04 * |
| High 4 | 5.00E-08 * | 1.24E-08 * | 3.33E-04 * | 1.00E+00 | 1.20E-08 * | 1.00E+00 |
| Low 2 | 1.52E-14 * | 1.52E-14 * | 2.13E-01 | 1.20E-08 * | 1.00E+00 | 7.26E-09 * |
| Low 4 | 3.86E-07 * | 1.03E-07 * | 1.76E-04 * | 1.00E+00 | 7.26E-09 * | 1.00E+00 |
|  |  |  |  |  |  |  |
| **Table S5: Tukey's HSD Pairwise Group Comparisons Test for As and Se Concentration in Plant Samples.** Groups were defined by their time post seeding (2 and 4) and treatment level (control, low, high). Kruskal-Wallis test was first completed and showed significant difference in As (p-value=7.67E-13) and Se (p-value=1.83E-14) concentration profile. (*) signifies that there was a significant difference between the groups (p-value>0.05) | | | | | | |
| **As** | | | | | | |
|  | Control 2 | Control 4 | High 2 | High 4 | Low 2 | Low 4 |
| Control 2 | 1.00E+00 | 9.99E-01 | 1.64E-03 * | 1.40E-05 * | 6.00E-04 * | 9.73E-03 * |
| Control 4 | 9.99E-01 | 1.00E+00 | 5.35E-03 * | 5.90E-05 * | 2.02E-03 * | 2.67E-02 * |
| High 2 | 1.64E-03 * | 5.35E-03 * | 1.00E+00 | 8.14E-01 | 9.99E-01 | 9.98E-01 |
| High 4 | 1.40E-05 * | 5.90E-05 * | 8.14E-01 | 1.00E+00 | 9.63E-01 | 5.68E-01 |
| Low 2 | 6.00E-04 * | 2.02E-03 * | 9.99E-01 | 9.63E-01 | 1.00E+00 | 9.66E-01 |
| Low 4 | 9.73E-03 * | 2.67E-02 * | 9.98E-01 | 5.68E-01 | 9.66E-01 | 1.00E+00 |
| **Se** | | | | | | |
|  | Control 2 | Control 4 | High 2 | High 4 | Low 2 | Low 4 |
| Control 2 | 1.00E+00 | 1.00E+00 | 8.41E-04 * | 6.00E-06 * | 6.49E-03 * | 2.67E-02 * |
| Control 4 | 1.00E+00 | 1.00E+00 | 9.77E-04 * | 8.00E-06 * | 7.38E-03 * | 2.98E-02 * |
| High 2 | 8.41E-04 * | 9.77E-04 * | 1.00E+00 | 8.14E-01 | 9.96E-01 | 9.28E-01 |
| High 4 | 6.00E-06 * | 8.00E-06 * | 8.14E-01 | 1.00E+00 | 5.32E-01 | 2.57E-01 |
| Low 2 | 6.49E-03 * | 7.38E-03 * | 9.96E-01 | 5.32E-01 | 1.00E+00 | 9.97E-01 |
| Low 4 | 2.67E-02 * | 2.98E-02 * | 9.28E-01 | 2.57E-01 | 9.97E-01 | 1.00E+00 |
